# Recognizing the quiet extinction of invertebrates

**DOI:** 10.1038/s41467-018-07916-1

**Published:** 2019-01-03

**Authors:** Nico Eisenhauer, Aletta Bonn, Carlos A. Guerra

**Affiliations:** 1 German Centre for Integrative Biodiversity Research (iDiv) Halle-Jena-Leipzig, Leipzig, Germany; 2 Institute of Biology, Leipzig University, Leipzig, Germany; 3 Department of Ecosystem Services, Helmholtz - Centre for Environmental Research – UFZ, Leipzig, Germany; 4 Institute of Biodiversity, Friedrich Schiller University Jena, Jena, Germany; 5 Institute of Biology, Martin Luther University Halle Wittenberg, Halle (Saale), Halle, Germany

## Abstract

Invertebrates are central to the functioning of ecosystems, yet they are underappreciated and understudied. Recent work has shown that they are suffering from rapid decline. Here we call for a greater focus on invertebrates and make recommendations for future investigation.

Invertebrates rule the world as we know it in terms of biodiversity and the functioning of ecosystems^1^. This is why scientists have repeatedly called to assess this essential part of biodiversity as well as its ecosystem effects^2^. In addition to conspicuous changes of ecosystems, such as the decline of charismatic vertebrate populations, the less obvious disappearance of many invertebrates^2,3^ also has dramatic consequences for the ecosystem services humankind depends on^2,4^. Recently, a report of alarming declines in invertebrate biomass^3^ has triggered broad public attention that is now also percolating into political discussion and decisions in several countries. As a consequence, new national and international biodiversity assessments, monitoring initiatives, and action plans are being discussed, and scientists are asked for guidance.

First cross-taxon comparisons indicate that biodiversity loss may be even more pronounced in invertebrates (e.g., butterflies in Britain) than in plants and birds^5^. These studies suggest substantial changes in invertebrate diversity and community composition that have been happening almost unnoticed and indicate that species may become extinct before we even know about their existence^6^. The Red List of Threatened Species of the International Union for Conservation of Nature (IUCN)^7^ is an important reference for the threat of species, but it is still heavily biased towards vertebrates, with invertebrates being particularly underrepresented (Fig. 1). Thus, a broader taxonomic base for threatened species assessments, adequately representing invertebrates, will facilitate more profound conservation and policy decisions^6^.

**Fig. 1:**
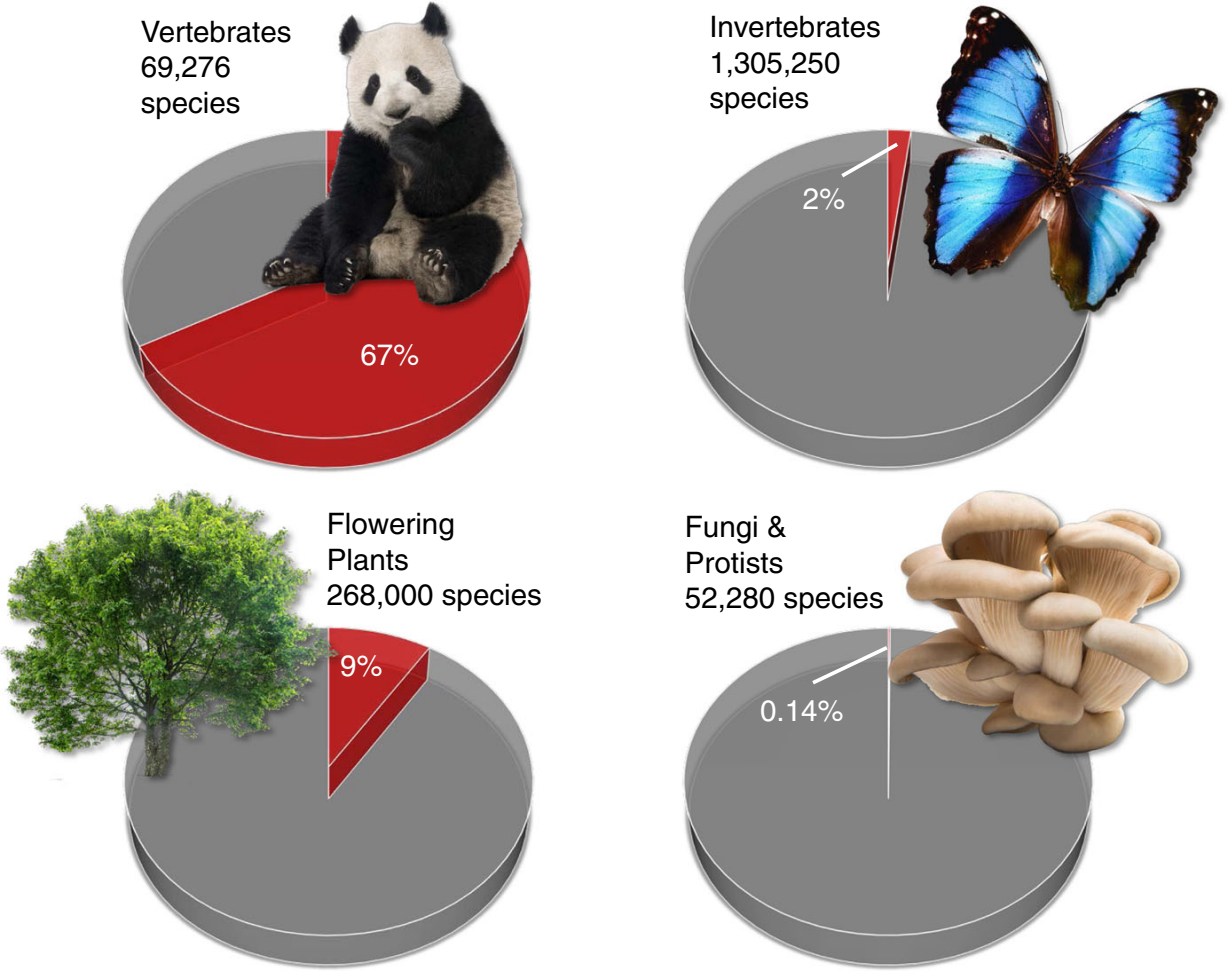
Underrepresentation of invertebrates on IUCN Red List. Examples for percentages of species assessed on IUCN Red List by 2018 in comparison to the number of described species^7^. Notably, there is high variability in the percentage of evaluated species within these broad categories. For instance, only ~0.8% of all described insect species was evaluated in 2018. Photo credits: panda: Eric Isselée; butterfly: Fotokon; tree: Production Perig; fungi: ksena32 (all Fotolia.de)

It is often the case in biodiversity assessments that there are spatial and taxonomic biases in available data, and this is especially true for invertebrates^4,8^. The majority of the invertebrate taxa that have received most attention in past biodiversity assessments is closely related to pollination. In fact, most animal pollinators are insects (e.g., bees, flies, butterflies, moths, wasps, beetles, and thrips), and bees are the most important pollinator group, visiting >90% of the leading global crop types^4^. Over recent years, public appreciation of pollinators has grown, and bees remain one of the better-understood taxa because of their important contributions to food security. The most recent assessment report of the Intergovernmental Science-Policy Platform on Biodiversity and Ecosystem Services on pollinators, pollination, and food production^4^ acknowledges that wild pollinators (mostly invertebrates) have declined in occurrence, abundance, and/or diversity. However, even for these widely-valued species, there are knowledge gaps, such as in regions outside of North-West Europe and North America.

These problems only become greater when other invertebrates are considered. While there is spatially and temporarily detailed data for some charismatic indicator taxa, such as butterflies in the European Union^9^, information about other invertebrates is lacking. For instance, soil invertebrates and soil-dwelling larval stages of flying insects, which represent a major biodiversity pool in terrestrial ecosystems, have been woefully neglected in many biodiversity databases and assessments, as well as in conservation actions and policies^8^. In addition, while assessments of invertebrate species richness, abundance^2^, and biomass^3^ provide important information regarding biodiversity changes, they may not capture more subtle yet ubiquitous changes in other biodiversity facets, including genetic, phylogenetic, and functional diversity and community composition.

## Monitoring biodiversity and ecosystem functioning

Invertebrates occupy many important trophic niches in natural communities^1^. Decreasing or changing invertebrate diversity and abundance can have strong effects on many ecosystem functions and services ranging from primary productivity, to pollination, and pest control. It adds to the complex picture that invertebrates can also contribute to human harm, e.g., mosquitos and ticks, which may have complex responses to climate change and habitat conversion. At the same time, many important invertebrate taxa that provide critical ecosystem services are still insufficiently represented in biodiversity monitoring. In fact, recent work has demonstrated that the diversity of soil invertebrates is of particular importance for the provisioning of multiple ecosystem functions and services across ecosystem types^10,11^, including soil erosion control and nutrient cycling.

As the need for improved monitoring of biodiversity becomes clearer, so does the need for comprehensive and widely-adopted strategies. Given that a major fraction of invertebrates lives below the ground, and considering their significant functional role^10,11^, biodiversity monitoring urgently needs to include soil organisms and functions^8^. Accordingly, biodiversity monitoring has to go hand in hand with ecosystem function monitoring to be able to recognize the functional consequences of changes in biodiversity. We have the appropriate tools at hand to monitor multiple ecosystem functions in a standardized way, e.g., through rapid ecosystem assessments of functions and ecological interactions that determine the functioning of ecosystems^12^.

Biodiversity and ecosystem function monitoring should be partnered with experimental validation of causal relationships and the exploration of process-based mechanisms. For instance, invertebrate effects can be studied under field conditions by manipulating their density and composition using exclosures^13^, and mesocosm laboratory experiments can be a promising tool to study multitrophic biodiversity-function relationships and the role of focal invertebrate taxa. However, there have been very few studies exploring the effects of higher trophic level invertebrates such as predators, and those that do exist have been performed almost exclusively in aquatic ecosystems.

Biodiversity monitoring also needs to consider multiple facets of biodiversity^14^, moving from focusing on the red list status or well-known species to analyzing functional traits (e.g., body mass, feeding type, trophic position, movement mode) and their roles in ecosystems. Accordingly, biodiversity metrics representing intra-kingdom and inter-kingdom interactions (type, network structures), genetic, taxonomic, and functional diversity should be considered. Furthermore, monitoring should address the complexities of spatial scale and wider landscape contexts, as well as the drivers of biodiversity change, such as climate change and land-use change, that may act at different spatial scales. Scientists should agree on representative and repeated sampling methods for different focal taxa and functions and how data from different spatial scales can be integrated to develop clear statements and recommendations for decision makers.

## Towards global collaboration on monitoring of invertebrates

Some of the most important and pressing scientific challenges are to appreciate the huge and partly undescribed biodiversity of invertebrates, and their importance for crucial societal benefits. This would require assessing their changes over time, identifying the main underlying drivers, and developing respective conservation actions. At the same time, the decline in taxonomic experts for invertebrates calls for urgent action for capacity building. Crucially, it requires international, interdisciplinary and transdisciplinary research consortia that guide future monitoring and the synthesis of past and future data. Here, it will be pivotal for different experts from academia, museums, natural history societies, and other NGOs, as well as government agencies to work together to draw on different knowledge domains.

We argue for a monitoring scheme that estimates invertebrate biodiversity changes that follows a threefold approach. First, existing data over the past decades needs to be mobilized, archived, and made interoperable to be analyzed for spatio-temporal trends. Here, innovative statistical methods allowing to integrate data of different spatio-temporal resolutions and qualities need to be advanced. Second, targeted resurveys of well-sampled sites will elucidate more in-depth trend analyses. Intersection with environmental data will allow for initial attribution analyses by inference that need to be followed by experimental studies. Third, new monitoring schemes need to be established at the national level. The spatio-temporal dimensions crossed with gradients of global change drivers may, however, lead to an explosion of sample size, and targeted gap analyses are needed to best design these new monitoring schemes and to optimize current ones. Since the causes and consequences of changes in invertebrate communities do not stop at country borders, different national biodiversity and function monitoring initiatives need to be harmonized within and across countries. Fostering capacities of taxonomic skills in society and academia, and jointly working with citizen scientists and volunteers will be pivotal to success. It is of equal importance to make use of recent advances in environmental monitoring, such as barcoding, environmental meta-barcoding, and (semi-) automated acoustic and video monitoring. An important initiative towards this goal is the Group on Earth Observations Biodiversity Observation Network (GEO BON), which aims to foster biodiversity monitoring across a range of Essential Biodiversity Variables by promoting the development of national and thematic biodiversity observation networks^14^.

The quiet and underappreciated extinction of invertebrates has important consequences for ecosystem function and human well-being. Novel biodiversity and ecosystem function monitoring initiatives are needed, and these require collaborative efforts from multiple sectors of society and innovative thinking to better understand and protect this significant portion of biodiversity. These will raise public awareness, increase scientific literacy of biodiversity loss, empower participants to support evidence-based decision making, and thereby also foster social and political innovation^15^ to combat invertebrate extinctions.
